# Rethinking ressentiment: democratic and urban implications

**DOI:** 10.3389/fsoc.2025.1573180

**Published:** 2025-10-16

**Authors:** Letizia Carrera

**Affiliations:** Department of Research and Humanistic Innovation, University of Bari Aldo Moro, Bari, Italy

**Keywords:** ressentiment, social envy, democratic promise, urban space, political participation

## Abstract

The article explores the complex interplay between resentment and democratic society, and the material and visible forms that this emotion takes within the city. Ressentiment emerge as key explanatory categories, influencing individuals’ perceptions and actions within social processes. Resentment is portrayed as a deeply democratic sentiment, arising from the perceived betrayal of the promise of equality inherent in democratic ideals. The article examines how this perceived injustice fuels a pervasive sense of resentment, which can either fragment social bonds or, alternatively, serve as a catalyst for political and social change. Urban space, with its dense and heterogeneous environment, is identified as a critical site where these dynamics become particularly visible. Cities, as synecdoche of society, not only reflect but actively shape social processes and collective feelings. They are arenas where perceived inequalities can either lead to social envy and resentment or foster solidarity and transformative activism. The article argues that addressing the roots and manifestations of resentment through inclusive and participatory processes is crucial for mitigating its destructive potential and harnessing it for positive social change. This approach involves creating urban spaces that facilitate critical reflection, social interaction, and collective action, thereby strengthening social and political efficacy among individuals and communities.

## 1 Introduction

Feelings and emotions represent an essential element in understanding the actions and choices of individuals, as well as broader social processes (Hoggett et al., 2013; Mesquita, 2022; Lindquist and Barrett, 2024). Emotions have long been the subject of study across various disciplines, from the humanities to neuroscience, including psychology and philosophy. Each approach has provided a unique perspective on understanding how emotions influence human behavior and social dynamics. From Darwin’s evolutionary theories, which view emotions as functional adaptations for survival, to cognitive theories exploring the role of thoughts in emotional processing, James-Lange’s peripheral theory focusing on the body’s physiological responses to external stimuli, and Cannon-Bard’s theory positing the thalamus as central in emotional processing.

Sociocultural theories, such as the neuro-sociological perspective, emphasize the influence of cultural contexts on the modulation of emotions, exploring not only how the brain processes emotions but also how social and cultural interactions shape neurological processes. This perspective aims to serve as an interdisciplinary bridge between neuroscience and sociology, seeking to understand how brain processes influence social behaviors and, conversely, how social dynamics shape brain structures.

Key researchers in this field include Warren TenHouten and Elizabeth Segal, who have significantly contributed to developing a theoretical framework exploring the complex interaction between the brain, emotions, and social behaviors. TenHouten, known for his pioneering work on the role of emotions in social processes, argues that emotions are not merely biological responses but serve as a crucial bridge between neurological processes and social dynamics^1^. Segal, on the other hand, delves into the role of empathy as the foundation of social connection, exploring its neuroscientific basis through mirror neuron research. She posits that empathy is a neurologically rooted process, yet one shaped by social and cultural experiences^2^.

Although this latter theory assigns significant weight to cultural and social elements, integrating a neuroscientific explanation risk shifting the focus toward individual and biological processes, which are not the primary concern of sociological reflection aimed at understanding broader processes. In this article, emphasis has been placed on the role of social processes, as well as social structures, norms, and values, in shaping mechanisms that generate resentment on both individual and collective levels. This leads to a discussion of how spatial dimensions—particularly urban space—play a decisive role within these social structures, thus shaping the conditions for a kind of spatialization of emotions, and more specifically, of resentment. And how resentment can have significant repercussions on the dynamics of democratic socio-political processes, with its implications taking shape within both the physical and symbolic space of the city.

The reflection seeks to understand how resentment manifests in reaction to perceived injustices, social inequalities, exclusion dynamics, or lack of recognition, rather than exploring how the brain processes emotions such as resentment on a neural level. Thus, deferring to more specific studies from equally necessary perspectives, the analysis focuses on resentment from the historical, political, economic, and cultural contexts that produce and sustain it—contexts that, under certain conditions, can transform resentment into a powerful force for change. This avoids any essentialist view of emotions, suggesting instead that resentment is not rigidly determined by biological processes but is a social construct, a dynamic outcome of historical and social conditions, and must therefore be understood within specific socio-spatial contexts.

Examples of this more analytical-qualitative approach, which forms the theoretical foundation of empirical analyses such as those by Banning (2006), Cramer (2016), Kurer et al. (2019), Ferrari (2021), Abts and Baute (2022), Melli and Scherer (2024) and many others, can be found in the philosophical analyses of Scheler (1915) and Girard (1981), as well as the sociological works of Barbalet (1992), Cattarinussi (2006), Fantini et al. (2013), Tomelleri (2004, 2010, 2023), and Smith (2020).

The analysis of the multiple theoretical approaches that connect emotions to both psychological and social dimensions go beyond the scope of this article^3^. Instead, it is article focused on resentment, examining its inherently social character, the social processes from which it originates, and the conditions under which it becomes a disruptive force in social bonds or, conversely, can acquire a prosocial significance.

The indispensable starting point is the relational dimension of resentment. As Stefano Tomelleri notes with regard to this specific emotion: “This convergence between emotional and institutional dimensions of social life is made possible by the fact that affective phenomena take the form of a social relationship, rather than that of an individual psychological phenomenon” (2024, 142).

The resentment should not be understood as mere individual characteristics or subjective reactions to external events, but as doubly social in nature—both because they belong to groups and communities, and because they are rooted in the internalized representations of individuals through their processes of socialization and in broader social dynamics.

This complex process becomes particularly visible in the city, where the individual and the social are connected by circular bonds (Cacciari, 2009), that, as Lefebvre (1974) intuited, is a synecdoche for society—a part that stands for the whole and where, as in any other socially produced space, social processes and themes take material form. The city is not merely a stage on which social processes are performed but plays an active role in shaping and guiding their dynamics. In this sense, it is in the urban space that collective emotions and among them resentment, take shape in an indispensable continuous reference between the different dimensions. The urban space is the site of experiences and can be a place of wonder and awe, anxiety and fear, solidarity, social envy, and ressentiment. It is the dense and heterogeneous city, where differences coexist in close proximity, that can easily become the place where perceptions of those differences and inequalities emerge, and where the connection between the individual conditions and the social models becomes most evident. When these differences are not addressed by public welfare policies, they ultimately end up consolidating into processes of social bond loss and individualistic drift, within which the sense of “relative deprivation” (Runciman, 1966; Boudon, 1986) becomes the measure of each individual’s un/satisfaction and un/happiness. In the absence of perceived ways out, social envy and the conviction of having suffered, or rather of currently suffering, an injustice, manifest as widespread ressentiment.

To examine these processes, this article conducts an analysis of the social nature of resentment, drawing on the authors who have explored it. This emotion is then connected to the principles of democracy and its (betrayed) promise of equality, an aspect that makes resentment one of the most political of emotions. Finally, the article discusses how resentment takes on spatial dimensions, manifesting in the city as a site of everyday life. It is precisely within this context that resentment reveals its dual nature, its ambivalent force, both as a paralyzing emotion capable of breaking social bonds and as a driving force for change.

This specific reflection on resentment thus opens up a broader discussion on the spatialization of emotions and on the potential of physical space as both a field and a condition of possibility.

## 2 The social nature of ressentiment

Even when ressentiment manifests as an individual experience, it inherently possesses a social nature, as Cattarinussi (2006) argues. Ressentiment arises from envy, which is tied to the comparison of one’s own condition with that of others who possess something different and socially desirable. “Envy precedes resentment not only from a psychological perspective, but also from a historical and social one”. Its nature as the outcome of inherently social processes lies in the triangulation articulated by Girard (1991): “subject-object-other subject,” evolves into ressentiment when there is an inability to attain the same status, coupled with an awareness of one’s powerlessness.

Referring to Nietzsche (1887) and Scheler (1915), the two main classical authors who addressed this sentiment, it can be observed that for both, despite their significant differences, *ressentiment* is tied to individual experience but is inseparably connected to one’s relationship with society. Recalling Max Scheler’s semantic clarification, “Ressentiment (written in italics throughout) (…) is marked by the unconscious transformation of envy, shame, or inefficacious anger of powerless and frustrated individuals into vindictiveness and hatred, compensating for a chronic perceived inferiority and deficiency to attain what one values or desires” (Capelos et al., 2021).

Nietzsche associates ressentiment with a specific category of individuals. The man of ressentiment is the weak, the fragile, the subterranean, the miserable, and the plebeian. When confronted with something perceived as unjust, “the strong” respond with action, while “the weak” settle for imagining a revenge that will not come by their own hand and is seen as the only means to overturn the relationship that currently subordinates them. In this scenario, truth is betrayed when the recognition of one’s weakness leads the resentful to invert values, turning the characteristics of the strong into negatives, perceived as personal threats^4^. The *mimetic desire* of the other can be observed through the words of Girard: envy towards those who embody the social model we wish to refer to, and the social group (of aspiration) we wish to belong to. When it results in failure, it generates resentment, to which the weaker individuals respond with an inversion of values, a denial of the other, masked behind a moral judgment (Tomelleri, 2010).

Max Scheler’s “provides a phenomenology of ressentiment that is decidedly more in-depth and detailed than what is found in the pages of Nietzsche’s “Genealogy of Morals.” It arises from a desire for a revenge, but it does not equate to it” (Colombetti, 2020, 40). The concept of resentment (ressentiment) refers to a deep-seated emotional response characterized by suppressed feelings of powerlessness and frustration, typically arising from a perceived injustice or inability to act against a real or imagined wrong. Unlike fleeting emotions such as anger or envy, ressentiment festers over time, as individuals or groups experience a chronic inability to overcome obstacles or address grievances. “it is a particularly violent tension between the development of the impulse for revenge, hatred, envy, on the one hand, and impotence on the other” (Scheler, 1975: 38). Prolonged frustration leads to the internalization of negative feelings, often manifesting in a desire for revenge or the moral inversion of values, where the traits of those perceived as stronger or more successful are denigrated or demonized. In Scheler’s view, ressentiment is not just an individual phenomenon but can permeate entire social groups, shaping collective attitudes and behavior, especially in contexts of social inequality or perceived powerlessness.

In Scheler’s view, ressentiment is not just an individual phenomenon views ressentiment as something that arises from a losing comparison, from a process of comparison in which one emerges defeated. This defeat gives rise to violent passions, which are, however, suppressed because, being socially weak, the individual is unable to act. This impotence results in a process where feelings of envy and powerlessness progressively infiltrate soul and being of the individual. From this perspective, ressentiment always has a social nature but penetrates the individual and eventually becomes part of the deep structure of their personality, rather than merely influencing their actions. As Vanni Rovighi writes, “there are no resentful acts, only resentful individuals” (Vanni, 2007: 50). “A self-poisoning of the soul, with well-defined causes and consequences, a permanent psychological state that arises from the systematic repression of certain emotions and affections that are normal in themselves and inherent to the fundamental structure of human nature (…) it is a particularly violent tension between the development of the impulse for revenge, hatred, envy, on the one hand, and impotence on the other” (Scheler, 1975: 3).

The core of this feeling is not merely in experiencing envy, but in the process of its obligatory sublimation in the face of lived experiences and a sense of social powerlessness. In this sense, ressentiment affirms its fully social nature: The extent to which ressentiment forms in entire groups and individuals is linked first to the predispositions of the human material in question, and second to the structure of the society in which they live (Scheler, 1915). There is thus a constant interplay between individual attitudes and collective normative models, and like all other emotions, ressentiment is not a purely intra-individual psychological content but rather the outcome of a relational dynamic that is historically and culturally situated and is continuously defined through interactions and relationships (Gordon, 1981).

In the framework of the socialization process, we learn values and normative models that become later latent, as thematized by Talcott Parsons, and these serve as the key to interpreting not only the social context and the processes that take shape within it, but also themselves. Ressentiment, therefore, is doubly social: it is defined in a comparative and relational key, and it depends on the social desirability of values, achieved goals, and resources possessed or not possessed. It is based on the relationship between what some individuals have and what others can only desire, giving rise to the mechanism of “relative deprivation”^5^ (Runciman, 1966; Boudon, 1986)—a situation in which individuals perceive a discrepancy between what they have and what they deserve or desire to have, in comparison to other individuals or groups of belonging, reference, or even aspiration. This feeling of deprivation is not necessarily linked to a lack of essential or absolute needs but rather to a subjective perception of disadvantage compared to prevailing social, economic, or cultural standards.

In the framework of the relational dynamics that inevitably characterize the social context (Simmel, 1908), resentment emerges as a *mimetic desire*, the desire “to be according to the other,” as Tomelleri observes when analyzing Girard’s reflections (Girard, 1991).^6^ It is, therefore, essential that the perception of oneself and one’s own identity be strongly connected to the belonging society and to the specific group to which individuals feel they belong, comparing themselves with the other members of that group (e.g., Tajfel et al., 1971; Brewer and Miller, 1984; Sears and Kinder, 1985; Tajfel and Turner, 1986). When what is perceived as a lack is accompanied by the individual’s experienced impossibility of activating processes to achieve the same results and attain the desired goals, they internalize the awareness of this impossibility. As previously noted, social envy transforms into ressentiment, a process that tends to radicalize moreover under conditions of uncertainty (Grieve and Hogg, 1999; Mullins et al., 1999).

In this perspective, resentment appears as one of the most profoundly modern emotions.

Ressentiment is the offspring of humiliation, an unfulfilled promise, an open yet simultaneously denied conflict, an unhealed wound, an obstructed desire for change, and a disappointed expectation of fraternity and solidarity. It is a feeling. The desire for revenge and the sense of social powerless they feel and suffer ends up trapping them in repressed anger, which can be either latent or manifest (Brown, 2018).

This powerless anger, incapable of generating change, arising from comparisons with rivals, and in this sense, it echoes the “*amour-propre*” described by Rousseau, whose distinguishing feature lies precisely in comparison (Tomelleri, 2004). However, while Nietzsche frames it as a class issue— a sentiment of “the weak” against “the strong”—Scheler sees ressentiment as a more pervasive emotion, one that also grips the bourgeois class. Although the bourgeoisie may not need to assert themselves, they are caught in a “competitive frenzy,” and as Bacon pointed out, even those in a socially advantageous position can still experience envy or ressentiment. This is because, when someone else advances, they may feel as though they are falling behind. This bourgeois extension of the dynamic described by the concept of “relative deprivation” spares no one, not even those with high levels of economic and social resources. It is always possible to compare one’s situation with that of those who “have more” and consequently feel in a state of relative disadvantage and unfulfilled desire.

The concept of *mimesis* can be valued ‘as a privileged interpretive key for a genealogy of resentment capable of connecting the inner sufferings of man with the broader transformations taking place in contemporary society. Even beyond the profound differences in various theoretical interpretations, ressentiment is thus defined within a relational logic—an intoxication of the soul that stems from looking at those who are different, or simply at the “other” (Kancyper, 2003). This social character becomes magnified when ressentiment takes on a collective dimension, as seen in relations between peoples or classes, arising from a sense of competition that remains perpetually frustrated in comparison to those who have more, or much more, of what one feels entitled to. Consequently, what others possess becomes the standard against which one’s own condition and desires are measured, generating a perception of one’s own inadequacy.

Ressentiment manifests as “the contemporary sentiment that oscillates, in a schizophrenic movement, between an exacerbated and narcissistic desire for individual affirmation and a deep and radical sense of sadness [and powerlessness]. (…) Ressentiment is the emotional state of someone who has long desired something, but has been unable to achieve it, and who feels they will never be able to realize what they had once imagined” (Ferro, 2007: 16).

## 3 Ressentiment and democratic space

This complex relational dynamic, within which ressentiment takes shape, acquires a particularly distinct force—seemingly paradoxical—in democratic societies, where the formal promise of equality is internalized by individuals but subsequently betrayed at the substantive level. “The root of conflict and violence is not found in the divergence of desires, but in their convergence, which follows a strict logic”. *Mimetic desire*, understood as an intrinsically relational and social notion, is deeply connected with the universalistic ethos of modern democracy. Ressentiment, therefore, is a deeply democratic sentiment, not only because, as Scheler observes, it can be experienced by anyone, regardless of class, but primarily because it arises from the betrayal of the promise of equality, which is the foundation of democracy. As previously mentioned, it is the result of the perceived gap between one’s current condition and the condition to which they feel entitled. Ressentiment does not necessarily stem from the objective lack of something but rather from the feeling that one lacks what others possess and believes they are rightfully entitled to. Castelfranchi writes, “the more a person sincerely limits their aspirations to what they can achieve or what is appropriate to their status, the less they will envy [and thus the less they will feel ressentiment]” (Castelfranchi, 1857: 148). An illustrative example is found in Charles Baudelaire’s depiction of “the eyes of the poor”^7^—eyes that gaze in fascination at what does not belong to them yet are not driven by any desire for what they lack or for a world to which they feel they do not belong. In their eyes, there is no ressentiment, only surprise, wonder, and awe, for they perceive that distance and difference as part of the *natural order of things*.

Ressentiment, on the other hand, is tied to the theoretical promise of equality, which is learned and internalized as a right but fails to materialize or be actualized in daily practice. It is the result of a disappointed expectation of fraternity and solidarity. For this reason, the society most laden with ressentiment is one where universal rights are formally combined with social equality, which in practice translates into significant disparities in power and access to material goods (Scheler, 1915). In this sense, democracy’s limitation lies in generating expectations of equality without providing concrete guarantees for achieving it, and more importantly, without even offering realistic pathways to realize such equality. It is within this space of unfulfilled promises that ressentiment is born.

The *ressentiment* is originated and intensified by the “competitive frenzy” described by Tocqueville, identifying it as a central ailment of modern society—a society that builds itself on the idea of equal individuals, where everyone believes they can access everything. In contrast to the idea of ressentiment as a sentiment of the defeated and the weak, Scheler presents it as an emotion that can also overwhelm the victors, manifesting as indiscriminate repressed anger stemming from competitive anxiety that is destined to be perpetually frustrated. This, as Tomelleri notes, is a “systemic dissonance” that goes beyond the condition of certain individuals and instead defines itself as a widespread process and structural feature of society itself. Ressentiment moves beyond the rational calculation of costs and benefits, taking shape instead in a kind of grammar of emotions and feelings, embedded in the relational knowledge found in rituals, habits, and routines. It becomes the price paid for lost dignity and the breakdown of cohesion in interpersonal relationships (Kancyper, 2003; Ansart, 2002).

The complex nature of ressentiment, therefore, arises from the fact that it is the evolving outcome of more or less structural processes and conditions. Firstly, the gap between one’s lived experience and the connected legitimate expectations and desires; secondly, the conviction that there is nothing that can be done to alter this perceived unjust condition. While the first could serve as a foundation for action aimed at improving one’s situation, its combination with the second emotion instead paralyzes individuals in a state of resigned bitterness. Thus, according to Scheler, ressentiment becomes the result of a misunderstood egalitarianism: the desire for equality and the impossibility of achieving it translates into the denial of the worth of those perceived as superior, or into a reversal of values themselves. This results in a purely negative ethic, where individuals turn in different ways against “the system” and its values, promoting a sectarian logic that denies intersubjectivity and dialogic interaction (even in its conflictual form). This dynamic creates closed groups with strong identity-based foundations, which do not propose a different “cosmos of values” but instead estrange themselves from the prevailing ethos—thereby inadvertently reinforcing its existence.

To understand how ressentiment operates, it is necessary to recognize the gap that can exist between the right to equality of conditions and the actual opportunities and possibilities provided by the cultural and social system to achieve this equality. As observed, the expectation of a legitimate right to equality underpins the profound connection between ressentiment and democracy, and, in this perspective, between ressentiment and modernity. Modernity, in fact, presents itself as both a premise and a promise of equality, yet simultaneously forces individuals to experience the dissonance between the rhetoric and formal declarations of equality and the actual and concrete conditions they encounter and experience in their everyday life.

As Harvey (2016) notes in his analysis, the capitalist model, that bourgeois modernity has ultimately absolutized and normalized, fundamentally violates and denies the conditions for a widespread right to a high quality of life within urban and social spaces. In these spaces, the failure and betrayal of the promise of equality unfold. The structural conditions stemming from the resources individuals possess—their economic, cultural, and social capital, as Bourdieu (1982) would describe, create deep disparities among people. The betrayal takes shape in the inability of public institutions to provide adequate and widespread opportunities (Nussbaum and Sen, 1993; Nussbaum, 2000; Sen, 1999)^8^ that might, at the very least, mitigate the weight of personal resources and differential advantages in ensuring substantive, rather than merely formal, equality of opportunities. This situation exacerbates the gap between individuals’ goals, based on the normative models to which they are socialized, and the actual means at their disposal to achieve them.

This condition corresponds to what Merton (1936, 1938) defined as “anomie”, referring to the gap between what individuals have been led to desire—and thus adopt as their goals—and the conditions provided for them to realistically achieve those desires and objectives—the means (Savela and von Scheve). It represents a systemic imbalance between the ends tied to the society’s normative and cultural models and the socially legitimate means available to attain them. The late-modern egalitarian society, equal in its proclaimed values but profoundly unequal in terms of power and access to material resources, creates a rift between what is legitimately desired and expected and what is actually experienced in daily life by individuals.

The modern democratic society is, therefore, a space where envy becomes a widespread and structural sentiment, transcending social classes and extending beyond the boundaries of the most deprived subjects Nietzsche had in mind. In this sense, as Girard (1981) observes, ressentiment corresponds to a specific dynamic of desire—one that arises from the imitation of another’s desire. In a more or less conscious way, individuals aspire *to be like the other*, and every personal failure as every success of the *other* is experienced as a personal narcissistic wound that magnifies the perceived distance.

In this sharp contradiction between expectations of equality and structural inequalities, the latter are often perceived and experienced as personal failures by individuals who, as Ulrich Beck keenly observed (Adam et al., 2000), are desperately seeking individual solutions to systemic contradictions. The right to individual self-realization (Lash, 2000) is transformed into the duty of self-realization, placing full responsibility for their fate onto individuals and generating a persistent fear of failure (Han, 2020). De Nardis (2020) notes that individuals suffer from the frustration of not achieving their goals due to obstacles and difficulties that are not easily overcome, yet they see others, within the same socio-environmental system and in seemingly analogous conditions, overcoming these challenges and achieving success. This situation gives rise to what Han (2020) describes as the “performance society,” centered on extreme competition. It turns daily life into a constant race—both metaphorical and literal—against others, perceived as competitors, in the pursuit of success, profit, and superiority over those left behind, who are unable to keep up with the pace and are doomed to fall behind the “professionals of the fast-paced world”.

“They are the ones who never stop, who work around the clock, the Stakhanovites of the world, those who, while you sleep, stay awake and gain ground, perhaps at your expense. They are never aflicted by nostalgia, laziness, or other unproductive emotions. They have become professionals of the world, while you remain an *amateur*” (Cassano, 2011, 161). In this context, speed has consolidated as the natural rhythm of life and has progressively become a normal feature of existence, shaping the figure of the “homo currens” (Cassano, 1996, 2001). This new subject, much like the blasé and cortical man, the result of intellectualization described by Simmel (1903), is both the outcome and active participant in the naturalization of speed as a condition for survival in a competitive scenario where the responsibility for success—and failure—rests entirely on the individual (Wacquant, 2010).

This *new type of individual* is particularly susceptible to ressentiment, which becomes the offspring of egalitarianism on one side and free competition on the other, embodying the dark side of the promise and desire for well-being. This ressentiment fuels latent hostility and feelings of disappointment and injustice, which in turn spark a desire for revenge against others (Tomelleri, 2010; Feather and Sherman, 2002).

The perception of injustice is heightened by the belief that, in an increasingly competitive context lacking equal opportunities, it is often not the most qualified but rather the most adaptable who occupy key positions, perpetuating systemic contradictions. This form of social Darwinism evokes an emotional response of indignation, based on the conviction that some people hold positions despite being unqualified for them (D’Urso and Trentin, 1990). The desired but unattained material and immaterial goods, seen through a competitive lens, generate a perpetual tension that leads to self-exploitation and a sense of inadequacy in meeting ever-growing competitive standards (Han, 2012).

## 4 The political dimension of ressentiment

On the political level, ressentiment reveals an essential duality. On the one hand, it can immobilize individuals in a social bitterness with no way out, breaking social bonds, undermining any sense of social solidarity, and transforming broader societal affiliations into attachments to homogeneous and closed sub-communities. On the other hand, ressentiment can also translate into a powerful, driving force for change.

Regarding the first dynamic, paraphrasing Mongardini (2009) in his discussion of the “solidary community of the fearful,” individuals may find themselves part of the “solidary community of the resentful.” In this context, ressentiment, like envy, is “not a progressive, innovative, or revolutionary force, because the socially envious individual does not think of equality as a social value but only of themselves: envy, therefore, cannot be a driver of change or democracy” (De Nardis, 2000a; De Nardis, 2000b: 61, 64). This gives rise to the political type of “angry citizens” (Capelos et al., 2022), who channel their ressentiment over a lack of opportunities and the perceived and experienced inequalities into a paralyzing emotion and a choice of “sterile lament.” This fuels social polarization and fractures the sense of cohesion, fostering and favoring populist tendencies through the channelling of feelings of ressentiment and frustration, creating a “us versus them” narrative (Demertzis, 2006; Eco, 2020; Hoggett et al., 2013). Albert Hirschman, in his work “The Passions and the Interests” (Hirschman, 1977), discussed ressentiment in the context of economics and politics, focusing on how emotions, including social envy and ressentiment, shape the economic and political choices of social groups, creating conditions for populism and revolts against elites (Mansbridge and Macedo, 2019; Manunta et al., 2022).

Democracy relies on active participation and the opportunity for citizens to express dissent and seek change through peaceful and institutional means. However, when large segments of the population feel themselves excluded, marginalized, or ignored, high levels of *ressentiment* can emerge. This emotional phenomenon reflects a condition in which social groups, unable to directly address or resolve perceived wrongs, develop feelings of frustration and bitterness that manifest politically. In this scenario, the conditions for common social goods are broken, as these are “a set of necessarily shared goods that enable the unfolding of social life, the solution of collective problems, and human coexistence” (Donolo, 2017), which “constitute the warp and weft of any social fabric, and thus of any community” (Manzini, 2018: 33). Furthermore, the concept of “social love,” known as agapic or civic love, is overturned or at least denied in its conditions (Araújo et al., 2015; Araùjo et al., 2016; Iorio, 2014, 2015, 2016; Cataldi, 2018; Cataldi and Iorio, 2018)^9^.

On the other hand, regarding the second dynamic, rather than leading to destructive conflict, ressentiment can have an evolutionary outlet. “Envy [and consequently *ressentiment*] in itself is not an evil to be eradicated. It can, rather, ‘change its sign.’ It is the driving force of competition and can be a reason for growth and progress”. Hatred, envy, and even resentment can generate moments and processes of rupture, but also alliances, collaboration, and friendship. The *mimetic desire* from which they come to life is therefore a relationship that can take on different social forms and initiate equally different processes. The experience of powerlessness can transform into a desire and possibility for changing structures and relationships through a reworking of the wounded memory, both individually and socially (Améry, 1987). In this perspective, ressentiment acts as a catalyst for regenerative change in the social and political system^10^. Ressentiment, therefore, can be transformed into a powerful transformative force, into a sentiment with a pro-social value, “a spring that can trigger the transformation of dissatisfaction and discomfort into projects, creative works, the realization of greater social justice, and fraternal shifts in daily vital relationships” (Tomelleri, 2010: 32). The rhetoric of equality, combined with increasing and ever more structural inequality with “no way out,” instead of generating separation and conflict, can translate into forms of political and social expression, avoiding a bitter retreat into oneself and instead becoming a driving force for change.

The vocalization of dissent and the pursuit of socially permitted forms of conflict can alleviate the sense of powerlessness in the face of perceived or actual wrongs, and potential ressentiment can become an opening toward the unknown. This transformation is anticipated by Lefebvre (1967) when he writes about *the right to a different city*, − “the *other city*,” one that emerges from the denaturalization of the existing order and the ability to envision a different space and way of inhabiting it. This alternative social space needs to be imagined and planned to be realized. Ressentiment can become the creative space that opens up new possibilities, offering itself as the symbolic space of politics and imagination, leading to what Bloch (1955) called “docta spes”. This is not mere hope, but an informed hope, based on an awareness of the dynamism of reality— a concrete force that helps build reality in a rational and forward-looking way.

When framed in this way, ressentiment can represent the possibility and impetus for something new, a chance to bring to light the stories of those who have lost and those who continue to lose.

The theoretical reference here is to the choice made by the so-called second generation of the Frankfurt School, who, combining the conflictualist matrix of their predecessors with a new openness to possibilities, turned their attention to the “resentful”—those living on the symbolic and sometimes material margins of society and social norms, such as women, immigrants, the unemployed, homosexuals, and other marginalized groups. These individuals, who experience deep ressentiment due to their unjust condition of inequality, represent the historical referents who are meant to receive the “message in a bottle” sent by the founders of the Frankfurt School^11^. The invitation is for these marginalized subjects to take on the role of being the driving force of change, drawing on their peripheral, lateral, and resentful perspectives, which are eccentric yet capable of bringing the social and cultural peripheries of the world to the center of political and social debate.

Among the political potentials of ressentiment—beyond its tendency toward bitter retreat into a present perceived as having no escape—there is also its generative force for change. However, ressentiment can also be used instrumentally by political forces to build their own consensus by fostering hostility toward other social groups, which are presented as the cause of the broader society’s discontent^12^. As Tomelleri writes, modern elites have sought to establish their economic and cultural hegemony by generating “an external and/or internal enemy on whom to pin the blame for their own failures. This has proven to be an effective way to transfigure the ressentiments generated by economic and social crises (…) a sort of self-regulation system for redirecting conflictual tensions toward new expressive forms of that sentiment” (Tomelleri, 2010: 17). This can lead to the consolidation of a new ideology of ressentiment, “as when, in the modern era, scapegoating (the enemy people, the threatening ethnic or religious minority) was justified and legitimized to give homogeneity to nascent national cultures” (Tomelleri, 2010: 21). This is the politics of ressentiment, a political culture in which political divides are rooted in our most basic understanding of ourselves, infusing our everyday relationships, and being used for electoral advantage by political leaders. Ressentiment thus becomes a tool to strategically ease the widespread sense of fear and uncertainty (Bauman, 2000, 2001), yet through the mechanism of scapegoating (Girard, 1981; Douglas, 1995) and the identification of types and categories of people onto whom blame and responsibility can be placed.

As Eco (2020) and Giovanni Sartori noted, this is a classic process of oversimplification, typical of populist political movements and trends. “The quintessential ‘other’ is the foreigner. (…) We are witnessing the power of fear over new waves of migration. By extending the characteristics of certain marginalized individuals to an entire ethnic group, Italy is currently constructing the image of the Romanian enemy, the ideal scapegoat for a society overwhelmed by a transformation, including an ethnic one, that it can no longer recognize itself in” (Eco, 2020: 12, 55).

Because of its complex political significance, the thematization of ressentiment within public discourse can serve as an indicator of the quality and direction of political reflection and the values underpinning it. Both the rhetoric of conflict and the absence of its thematization serve as indicators of systemic distortion, representing a limitation in the culture and dynamics of politics at the social level^13^.

## 5 The dual nature of ressentiment and the city

It is within space, as Max Scheler noted, that *ressentiment*—woven between the individual and social planes—takes material form. This sentiment emerges most acutely in cities, which symbolize progress, modernity, and the concentration of economic and cultural power and differences. In the urban environment, ressentiment surfaces both among citizens who feel excluded from the benefits of city life and among groups that view the city as a symbol of social inequality. In the urban space, people meet, see, and compare themselves to one another, becoming acutely aware of their differences and how opportunities are often accessible only to those who already possess high levels of cultural, economic, or social resources. This dynamic can create a sense of exclusion or disadvantage for some social groups, despite sharing the same geographical space (Carrera, 2024b). The proximity fostered by urban space can amplify both the scope and intensity of ressentiment, sometimes culminating in protests. Examples include Pride marches asserting the right to chosen identities, Family days advocating for traditional definitions of family, strikes, demonstrations by the “Sardines” movement, protests by the “tent movement,” and the actions of the Gilet jaunes, and so on^14^. This physicality of contestation and conflict can be interpreted either as a sign of a divided or even fragmented society, where individual or corporate grievances take precedence, or, in line with Hirschman’s (1970) categories, as a vocalization of dissent and an adoption of “voice strategies”. This political action transforms ressentiment from a sterile feeling that fractures social belonging into a driving force for change, by regulating conflict and, paradoxically, reaffirming a sense of belonging to society. An example of transformative ressentiment is the Italian movement of Sardines. Emerging at the end of 2019 as a spontaneous response to the rise of sovereign populism in Italy, the Sardine movement represented an original and unexpected form of political mobilization. Without a party, flags, or traditional leadership, thousands of individuals gathered in Italian squares—reconfigured as third spaces—to reaffirm a vision of inclusive and participatory democracy. This act of convening in urban space was not merely logistical but profoundly symbolic: the square re-emerged as the quintessential political arena, where bodies and voices came together in opposition to divisive and aggressive rhetoric, restoring meaning to collective presence in public space.

Another example of the urban political canalization of transformative power of resentment is represented by the Gilets Jaunes (Yellow Vests) movement, which emerged in France in late 2018, epitomizes a grassroots mobilization rooted in economic and social grievances, particularly among residents of rural and peri-urban areas. Initially sparked by a proposed fuel tax increase, the movement rapidly expanded to encompass broader discontent with socioeconomic inequalities and perceived governmental neglect. A distinctive feature of the movement was its occupation of roundabouts and public spaces, transforming them into arenas of protest and community engagement. These spaces, often located in peripheral regions, became symbolic sites where citizens could express their frustrations and demand recognition. As noted by scholars, the spatial dynamics of the Gilets Jaunes involved a dual strategy: localized actions combined with national demonstrations, effectively bridging the gap between marginalized areas and urban centers. The movement’s horizontal structure and reliance on social media platforms facilitated decentralized organization and communication, allowing for a diverse range of voices and perspectives to be heard. This structure also enabled the transformation of individual resentment into collective action, fostering a sense of solidarity among participants. As observed in analyses of the movement, the Gilets Jaunes harnessed shared grievances to galvanize political participation across various strata of society. In essence, the Gilets Jaunes movement illustrates how urban and rural spaces can be reimagined as platforms for political expression, where resentment is channeled into active engagement and demands for systemic change. The movement underscores the potential for spatial practices to serve as catalysts for democratic participation and highlights the importance of inclusive public discourse in addressing societal challenges.

As Henri Lefebvre reminds us, space is always socially produced. In this light, the Sardines re-signified Italian piazzas as spaces of community, dialogue, and civic resistance. At the emotional core of the movement lay, at least initially, a widespread sense of resentment, a discomfort with hate speech, political oversimplification, and the perceived erosion of democratic values. What sets the Sardines’ resentment apart, however, is that it did not remain passive or destructive. Following a logic akin to that described by Nussbaum (2016), resentment was transformed into a political emotion, capable of mobilizing energies toward a form of active and inclusive citizenship.

A key feature of democratic urban culture is the drive toward a complex and mature form of equality—one that recognizes the profound differences among individuals in terms of needs, expectations, desires, and resources, and their right to have those differences acknowledged or compensated for when unwanted. When politics, charged with mediating between different and sometimes conflicting demands, fails in this task and disappoints expectations, conflict can become visible in the city (Amendola, 2010). It may take the form of blind rage, stemming from social envy and a sense of being trapped with no way out, or it may emerge as a political protest that, while challenging the status quo, simultaneously affirms a sense of belonging to the social and political community.

As Lefebvre (1967, 1974) wrote, space—and within it, urban space and cities—materialize the normative models and processes that run through society. Cities are the places where these become most visible. Lefebvre, exploring the production of urban space, argued that modern cities are designed and developed to serve the interests of capital rather than ordinary citizens, seeing cities as places of alienation for workers and subaltern classes whose right to the city is eroded by dominant economic and political forces. Expanding this idea, neoMarxist geographer and theorist David Harvey explored the relationship between urbanization and capitalism, describing the city as the primary battleground for social and economic inequalities. He framed the city as a space where the conflict between capital and labor is most apparent, with the *ressentiment* of working classes or marginalized groups becoming a central element in the struggle for the right to the city^15^.

*Ressentiment* emerges as a reaction to the perceived loss of control over urban spaces^16^. As noted, ressentiment is generated within a cultural horizon of societies that are “increasingly oriented towards offering infinite possibilities of choice, but incapable of promoting the conditions of equal opportunity necessary to realize them” (Tomelleri, 2010: 32). This process particularly takes shape in the city, which represents a non-neutral space where density and diversity—its defining characteristics (Simmel, Park, and later Sennett, Sassen, Harvey, Amendola, Mela)—lead individuals to experience their differences as a daily reality. Urban peripheries, or areas marked by peripheral characteristics, and their inhabitants, may experience in a particularly acute way the politically dual nature of ressentiment. They may either retreat into a sterile social envy that fractures social bonds and fragments society, or they may activate forms of vocal dissent and protest that strengthen social solidarity within the communities or sub-communities to which they feel they belong. In the city, it becomes possible not only to be, but above all to show one’s resentment. To communicate, through the physicality of bodies within the materiality of urban space, the feeling stemming from the belief of having suffered an injustice, making protest and the places where it takes shape a means of social communication and a possible, albeit difficult, dialogue.

As previously observed, ressentiment is a deep-seated bitterness toward a situation perceived (Thomas and Thomas, 1928)— and often experienced—as being without exit or solution. A key factor in the genesis of this perception is a lack of trust in politics and political leaders at various territorial levels, who are seen as distant from local communities and uninterested in protecting broad-based interests, often giving rise to populist trends (Demertzis, 2006).

One of the necessary strategies to attempt to regain the sense of perceived self-efficacy and institutional trust, in order to mitigate the destructive potential of *ressentiment* for both individuals and communities, is the activation of some structured and institutionalized wide participatory processes (Segatti and Vezzoni, 2007). These processes have two communicative and symbolic impacts: on one hand, they signal that administrations are genuinely interested in involving individuals in shared pathways of listening and co-designing urban and territorial policies; on the other hand, they create conditions for communities to feel the centrality of their civic and political role, allowing them to build upon their everyday experiences, thematize these experiences, and use them as the basis for concrete political proposals.

As Eco (2020) reflects, democratic politics should find ways to channel frustrations and feelings of powerlessness into forms of inclusive participation that are perceived as fair, thus preventing *ressentiment* from becoming a destructive force, particularly in the hands of populist or authoritarian leaders. Restoring political agency to individuals can help reduce their sense of powerlessness and increase their perceived political self-efficacy, which is inversely correlated with ressentiment and the alienation from a shared destiny.

So that participation is more than more than mere rhetorical strategy or a formal requirement (Carrera, 2025), it is necessary to create the material conditions that enable formative processes aimed at enhancing the capacities of citizens and communities (Montoya et al., 2000). To this end, specific and non-specific training programs for participation are essential to prevent the most socio-economically and culturally deprived citizens from being relegated to a state of political and social *silence*. From this perspective, educational pathways are essential for empowering people to imagine something different—Lefebvre’s *other city*—countering the perceived impossibility of realizing diversity, as lamented by Marcuse (1964), and going beyond what De Carlo (2015), echoing Domenico De Masi’s analyses, referred to as “the issue of the tile”, or the inability to envision something different from what already exists, which defines the only horizon of knowledge^17^.

For this complex goal, which cannot be achieved without the activation of multi-actor and multi-level networks connecting institutions and public administrations at various territorial levels, associations, the educational system, and individual citizens, the territorial dimension is an essential reference (Williams, 2019). “Participatory processes require the creation of inclusive spaces where citizens [and various territorial actors] can share knowledge, perspectives, and experiences to address complex and controversial issues” (Nabatchi and Leighninger, 2015). For this specific objective, it is crucial to have adequate public spaces that ensure inclusivity, functioning as “third spaces.” These are new, somewhat liminal and interstitial spaces that offer opportunities for rest, encounter, and recognition—serving as a platform for the collective social construction of creative responses to social change processes, starting from the urban space (Lefebvre, 1974; Bhabha, 2001; Soja, 1996, 2007). The concept of Third Space, introduced by postcolonial theorist Homi K. Bhabha, refers to an interstitial area where different cultures intersect, giving rise to new, hybrid identities. In Bhabha (1994), describes this space as a “site of enunciation,” where cultural differences are not simply juxtaposed, but actively negotiated, generating new and contextual meanings. This process challenges essentialist notions of cultural identity, emphasizing its fluid and continually transforming nature. However, some scholars have critiqued the concept for overlooking material inequalities and persistent power dynamics. For instance, Abou-Agag (2021) argues that while the Third Space promotes cultural negotiation, it can also be co-opted by neocolonial forces—such as international organizations and global networks—that sustain exploitation under the guise of equitable cultural exchange. In the urban context, geographer Edward W. Soja extended the concept to spatial theory. In Thirdspace: Journeys to Los Angeles and Other Real-and-Imagined Places (Soja, 1996), Soja explores how urban spaces are produced through social practices, representations, and imaginaries, becoming potential sites of resistance and possibilities for new forms of social justice. In sum, the Third Space is a powerful lens for understanding contemporary cultural and spatial dynamics. It highlights how identities and spaces are continuously negotiated and reconstructed, offering opportunities for transformation while also revealing the risk of appropriation by existing structures of power.

In this sense, third spaces are key elements of contemporary urban culture, emphasizing the role of space itself and the centrality of its symbolic and functional representation. They create opportunities for relationships, initiatives, and cultural and political exchanges between diverse individuals, potentially leading to more enduring associations (Carrera, 2022). As Amin and Thrift (2002) argued, the quality of the urban habitat can and should be cultivated in micro-public spaces, conceived as opportunities for cultural contamination practices that offer structured opportunities for ongoing discussion among different actors.

These local processes of participatory urban planning, self-organization, and forms of urban activism can contribute to triggering virtuous processes of critical reflection, while at the same time reinforcing a sense of belonging, social responsibility, and political engagement with their territories, and increasing their capacity to influence ongoing processes (Bobbio, 2005; 2019; Pellizzoni, 2005; Manconi and Porcaro, 2015). Through these paths, one can attempt to reduce the sense of powerlessness that causes perceived differences to translate into resentment, fueling individualistic choices and contributing to the weakening of social bonds.

## 6 Concluding notes

Using ressentiment as a lens for understanding social processes allows us to grasp both the foundational nature of the promise of equality that shaped modernity, and the inherent difficulties of this political and social project. These difficulties, stemming from an increasing mimesis and the widespread convergence of desires, as well as the conviction of having full entitlement to them, are translated into a kind of systemic impossibility within the neoliberal model, which on one hand denies the conditions necessary for the full realization of this possibility, and on the other hand places the burden of individual destiny entirely on the subject. This betrayal forms the space where ressentiment develops—stemming from both the impossibility of achieving a condition socially presented as the only desirable one for a successful life and the inability to escape or resolve this unattainable goal. This dual condition of powerlessness becomes social envy, bitterness, the denial of social virtues, and the toxic ressentiment described so sharply by Scheler, a materialized socio-spatial condition within the urban fabric, wherein the city emerges as a site of disillusionment and the crystallization of social discontent.

The difficult and necessary challenge should be addressed on two levels.

First, there is the broader cultural level, where it is essential to redefine the meaning of the “performance society” (Han, 2020) and the weight of values such as success, the identity value of consumption and competition. This requires moving beyond the representations of *others* as competitors and alleviating the fear of never measuring up to existential challenges. “Fear that, despite work and sacrifices, they are not able to maintain or attain the standard of living and social status they have previously enjoyed or aspire to” (Flecker, 2007: 41–42). Ressentiment plays a key role in shaping individuals’ social and political choices and how it feeds back into and reinforces the very worldview that generates it. This contributes to deepening the sense of division between “us” and “them.” It can drive the breakdown of social bonds, where instead of empathizing with those who are struggling, they are blamed, further reinforcing the process of individualizing responsibility. So it could be possible facing the “democratic dilemma” highlighted by Celis et al. (2021) that involves recognizing frustrations and grievance while maintaining hope and sustaining democratic values and ideals, supporting processes aimed at strengthening social virtues and ensuring the conditions for widespread democratic socio-political decision-making.

Second, on the more material level, there is the need to create physical conditions that provide opportunities to share and design change of the conditions in the currently dominant normative model. Starting from a deep reflection on welfare models capable of mitigating differences in terms of resources by intervening in a compensatory way, and on political community, as the result of reflections and the problematization of identity closures or, rather, of projective openings.

These two levels are interconnected by indispensable osmotic links, catalyzed by participatory processes that take shape in both the material and symbolic spaces of cities, particularly in third spaces. These spaces represent the arenas where change can be considered, once again, as possible. They are the result of social and political choices, while also providing opportunities for further change. Structural change should go beyond the logic of consumption and the measurement of life in economic terms, which divides individuals into winners (a few) and losers (many, if not most). The goal is not simply to guarantee everyone the same opportunities to pursue a lifestyle that is destructive to individuals and to the planet, but to rethink the ultimate goals and criteria for defining what is desirable, while building the cultural and material conditions to achieve these aims.

But, in this complex and changing scenario, cities reaffirm their central role by being both symbolic and material spaces where broader social processes are most visible. They function as dual spaces, capable of both generating high levels of ressentiment and defusing its destructive potential by channeling it into participatory forms of political and social change. Public spaces as squares and streets, where ressentiment is experienced and takes concrete form, manifesting as action, sometimes violent, explicit rupture, or revolt, but also as potential spaces of awareness and social and political reimagining. In this perspective, activating refined and consolidated *voice* pathways within structured and widespread participatory processes represents an antidote to social fracture and the loss of political self-efficacy among individuals. Thus, cities—borrowing from the title of D’Antonio and Testa’s volume (D'Antonio and Testa, 2021)—can be part of the solution in creating tangible conditions for equality or, at least, a real, tangible attention to this issue, functioning as a regulation mechanism of ressentiment.

From ressentiment representing a paralyzing emotion, generating “angry citizens” (Capelos et al., 2022), where *ressentiment* is seen as a key emotional mechanism in grievance politics^18^, to ressentiment as a propulsive force capable of generating change, triggering processes that lead to social cohesion (Ivarsflaten, 2008; Kurer et al., 2019; Carrera, 2024b).

In this context, future research could benefit from examining the relationship between groups differentiated by gender, age, or geographic location (such as urban centers versus suburban areas). Given its essential dual nature, one possible starting point for addressing the challenge of ressentiment is to thematize it, thus transforming it into a potentially (re)generative force for political change, aimed at (re)building social virtues and a sense of social and political responsibility for inclusive community with a forward-looking vision (Carrera, 2024a).

## Data Availability

The original contributions presented in the study are included in the article/supplementary material, further inquiries can be directed to the corresponding author.

## Footnotes

^1^He suggests that complex emotions such as shame, guilt, and pride are mediated by specific brain structures and that their manifestation is influenced by cultural and social contexts. Ten Houten’s theory allows us to view emotions not only as individual experiences but also as tools through which individuals assert themselves and reinforce social expectations and constraints.

^2^The ability to understand and share others’ emotions is essential for building strong social bonds and fostering cooperation. Segal applies this theory to the field of social work, demonstrating how a deeper understanding of the neurological foundations of empathy can enhance interventions aimed at improving individual and collective well-being.

^3^Emotions are at the center of psychological, social, and neurological theories, whose analysis extends beyond the scope of this article. Instead, this work aims to offer a theoretical reflection on the influence that a specific emotion— resentment, or “*ressentiment*” in its manifestation as a social emotion—can have on democratic political dynamics and its implications for urban space.

^4^Nietzsche used the term “*ressentiment*” to explain how Christian morality developed in opposition to aristocratic morality, transforming values associated with weakness (humility, poverty, submission) into virtues, while demonizing those of the aristocracy (strength, success, domination).

^5^*Cf.* Th. J. Scheff, “Looking-Glass Self: Goffman as Symbolic Interactionist.” In Symbolic Interactionism. 1985, 28(2), pp. 147–166. Regarding Relative Deprivation Theory, see Walter Runciman, Relative Deprivation and Social Justice. Routledge & Kegan Paul, 1966.

^6^“According to Girard, our desire is always a *desire to be according to the other*. The Girardian actors are a mimetic actors, driven by a passionate admiration for the other, who, in their eyes, assumes the role of a mediator. The life of each of us is the story of our relationships with our models, with the people we deeply desire to be, often secretly, and for this reason, we imitate them in our gestures and style. But this is not a simple, repetitive, and passive imitation of the other; rather, it is an active, reciprocal, and often even conflictual imitation.

^7^“(…) Right in front of us, standing motionless on the road, was a man in his forties, with a tired face and graying beard, holding a child by the hand and carrying another little one, too weak to walk, in his other arm. He was acting as a nanny, taking his children out for a bit of fresh air in the evening. All three of them were in rags. Their faces were extraordinarily serious, and their six eyes gazed at the new café with equal admiration, though with different shades according to their age. The father’s eyes seemed to say: ‘How beautiful! How beautiful! It looks as though all the gold of the poor has gathered to decorate these walls.’ The child’s eyes: ‘How beautiful! How beautiful! But this is a place only for people who are not like us.’ As for the youngest child, their eyes were too mesmerized to express anything other than a deep, dull joy. Songwriters often claim that joy softens the soul and tenderizes the heart. As for me, that evening, the song was right. Not only was I touched by that family of eyes, but I also felt a twinge of shame for our glasses and carafes, which were far larger than our thirst. I turned my gaze to you, my dear love, to find the same thought in your eyes, as I dove into those beautiful, strange, and sweet eyes of yours—your green eyes, full of whimsy and inspired by the moon—when you said to me, ‘These people, with their eyes wide open like doors, are unbearable to me! Couldn’t you ask the maître to have them removed from here?” (Charles Baudelaire, The Eyes of the Poor, in “Paris Spleen”, 1855-1864).

^8^The concept of “opportunities” is central to the Capability Approach developed by Amartya Sen and Martha Nussbaum. This approach focuses on what individuals are actually able to do and to be—their real capabilities—rather than merely on the resources or goods they possess. In this context, opportunities represent individuals’ true freedoms to pursue and achieve the ways of living that they have reason to value.

^9^The concept of social love, also known as agapic or civic love, refers to a form of love that extends beyond intimate personal relationships, reaching into the social and community sphere. It is characterized by key traits such as interdependence, solidarity, social justice, empathy, and respect. As such, it can be seen as a driving force for positive social change, capable of transforming social and political structures to make them more humane and just.

^10^“At the root of resentment, both in individuals and social groups, there is always a wound, an experienced violence, an affront, a trauma [to which] the person who feels victimized cannot react, due to their powerlessness. They ruminate on their revenge, which they cannot carry out, and this torments them relentlessly. Until it explodes. (…) What gives new strength to the oppressed, promoting revolt, revolution, or even regeneration” (Ferro, 2007: 14).

^11^The metaphor of the “message in a bottle” represents the attempt to communicate critical messages or truths in a social and cultural context where such messages risk being ignored or lost. In a society dominated by mass media and the cultural industry, critical voices struggle to reach a broad and receptive audience. This metaphor implies a mix of hope and despair: the sender hopes that someone in the future will find and understand the message, even though they are aware that the chances of this happening are slim.

^12^“Striking examples of how an emotion with a negative connotation, like hatred, can be promoted and prescribed by certain social groups are Nazi Germany or certain white communities adhering to the Ku Klux Klan. In both cases, hatred of Jews and Blacks becomes the appropriate emotional behavior, as its negative aspect is socially anesthetized. The ethic of success upon which contemporary Western culture is founded similarly employs emotions such as envy in a functional way, using them as motivational drives for the individual” (Cattarinussi, 2006: 41).

^13^“A given community will attempt to promote those emotional expressions that are functional to maintaining the socially established moral order, while at the same time seeking to penalize and remove those emotions that are antithetical and in opposition to the moral order [chosen as their own]” (Gius and Zamperini, 1995: 11).

^14^Other examples include the “Occupy Wall Street movement,” which highlighted the *ressentiment* toward the power concentrated in the hands of financial elites who control major cities like New York, expressing sharp criticism of economic inequality and the appropriation of urban space by financial institutions. Similarly, the riots in the banlieues of large French cities, such as Paris, have been driven by the *ressentiment* of immigrant communities and the lower classes, who feel excluded from the country’s economic and cultural life, responding to their sense of isolation and marginalization with unrest. Protests against gentrification in cities like Berlin, San Francisco, and Milan also reflect the struggle of long-standing residents to defend their neighborhoods and communities against urban transformations that exclude them.

^15^Harvey (2016) further pointed out how urban neoliberalism exacerbates these tensions by privileging private investment and real estate speculation at the expense of the needs of the population.

^16^Gentrification, a common phenomenon in global cities, often intensifies *ressentiment*. Working-class neighborhoods are transformed to attract wealthier residents, resulting in the displacement of the original inhabitants. This process is perceived as a form of cultural and economic expropriation, fueling *ressentiment* toward newcomers and the institutions that promote such changes. Historically working-class districts are altered through investment and real estate development, leading to the influx of wealthier residents and the expulsion of original inhabitants who can no longer afford to live there. This is often seen as an injustice and can be viewed as a form of internal colonialism, where spaces considered “authentic” or “traditional” are privatized and transformed to benefit a few, at the expense of the local community.

^17^“Asking people without experience what kind of neighborhood houses they want leads to predictable answers: namely, the house of the local doctor in Terni—essentially a worker’s house, but slightly larger, with more tiles, and a few extra gadgets” (De Masi, 1995: 66–67). This idea gave rise to the first exhibition aimed at enriching the imagination and showcasing the possibilities of architecture, precisely to overcome what is referred to as “the tile problem.”

^18^Ressentiment transforms the inputs of grievance politics—deprivation of opportunity, injustice, shame, humiliation, envy, and ineffectual anger—into anti-social outputs of morally righteous indignation, destructive anger, hatred, and rage.
